# Conbercept for Treatment of Neovascular Age-Related Macular Degeneration and Visual Impairment due to Diabetic Macular Edema or Pathologic Myopia Choroidal Neovascularization: A Systematic Review and Meta-Analysis

**DOI:** 10.3389/fphar.2021.696201

**Published:** 2021-10-12

**Authors:** Pengxiang Zhou, Siqian Zheng, Ente Wang, Peng Men, Suodi Zhai

**Affiliations:** ^1^ Department of Pharmacy, Peking University Third Hospital, Beijing, China; ^2^ Institute for Drug Evaluation, Peking University Health Science Center, Beijing, China; ^3^ Department of Pharmacy, Beijing Tongren Hospital, Capital Medical University, Beijing, China

**Keywords:** conbercept, neovascular age-related macular degeneration, diabetic macular edema, pathologic myopia choroidal neovascularization, anti-VEGF

## Abstract

**Background:** Conbercept is a new anti-vascular endothelial growth factor (VEGF) drug. Here, we systematically conducted the efficacy, safety, compliance, and pharmacoeconomic evaluation of intravitreal conbercept (IVC) compared with other treatments in patients with neovascular age-related macular degeneration (nAMD), diabetic macular edema (DME), or pathologic myopia choroidal neovascularization (pmCNV).

**Methods:** Databases of PubMed, Embase, Cochrane Library, ClinicalTrials.gov, SinoMed, China National Knowledge Infrastructure, and WanFang Data were systematically searched from the inception to July 27, 2021. Randomized clinical trials and pharmacoeconomic studies comparing IVC with control groups in adults with nAMD, DME, or pmCNV were reviewed and selected. Meta-analyses were performed using the fixed-effects model when pooled data were homogeneous. Heterogeneous data were analyzed using the random-effects model. Primary outcomes included visual improvement rate, mean change in visual acuity or best corrected visual acuity, and pharmacoeconomic outcomes. Additional outcomes were the mean change in fundus examination values, adverse events (AEs), quality-of-life measures, and number of injections.

**Results:** Among 3,591 screened articles, 22 original studies with 1,910 eyes of patients were finally included. For nAMD and DME, IVC was significantly associated with better visual acuity or best corrected visual acuity improvement and fundus quantitative measures than placebo, laser photocoagulation (LP), or intravitreal triamcinolone acetonide (IVT). However, IVC showed non-inferior efficacy to intravitreal ranibizumab (IVR) according to low quality of evidence, and there was lack of trials comparing the priority of IVC to other anti-VEGF regimens. No definitive increased risk of ocular or non-ocular AEs were observed in the study groups. All patients with AEs recovered after symptomatic treatments, and no severe AEs occurred. Patients treated with IVC might have higher quality-of-life scores than those in IVR in nAMD or LP in DME. Additionally, IVC showed cost–utility advantages in nAMD and cost-effectiveness advantages than IVR in pmCNV in China.

**Conclusion:** IVC is well-tolerated and effective for improving vision acuity and quantitative measures in fundus condition in patients with nAMD and DME compared with LP, IVT, and placebo, but gains comparable efficacy to IVR. However, well-designed, large-sample, and long-term evaluation of IVC shall be conducted in additional studies worldwide.

## Introduction

Age-related macular degeneration (AMD) is a degenerative disease of the macular region of the retina and has become a leading cause of severe irreversible vision impairment in people over 40 years worldwide. The number of individuals affected by advanced AMD has reached approximately three million by 2020 (Lim et al., 2012; Flaxel et al., 2020a). AMD is usually classified broadly into dry (atrophic type) and wet types (neovascular or exudative type). Although neovascular AMD (nAMD) accounts for the minority of confirmed cases, it is the main cause of severe central vision loss (Gottlieb, 2002). Diabetic macular edema (DME), a common complication of diabetic retinopathy resulting in vision loss (Bandello et al., 2017; Flaxel et al., 2020b), is defined as retinal thickening and edema involving the center of the macula. DME also plays a primary role in adult blindness and affects approximately 21 million people worldwide (Yau et al., 2012). Additionally, pathologic myopia, with the complications of choroidal neovascularization development (pmCNV), is also a leading cause of visual impairment (Ohno-Matsui et al., 2018). With the global aging and the increased prevalence of diabetes, nAMD, DME, and pmCNV have become severe global health issues with substantial socioeconomic implications.

Recent studies on the pathogenesis of nAMD and DME indicated anti-vascular endothelial growth factor (VEGF) played a vital role in preventing blindness, and VEGF inhibitors served as the first-line standard-of-care for patients with nAMD, DME, and pmCNV (VEGF, 2015). Compared with standard treatments, such as photodynamic therapy and laser photocoagulation (LP), intravitreal injection of VEGF inhibitors may exhibit superior outcomes in nAMD and DME treatment (Mitchell et al., 2018; Kim et al., 2019).

Ranibizumab, the first anti-VEGF drug in the field of ophthalmology, was approved for nAMD by the U.S. Food and Drug Administration in 2006, followed by aflibercept. In 2013, a new anti-VEGF agent conbercept (KH902) was approved by the Chinese National Medical Products Administration for the treatment of nAMD, DME, and pmCNV. Conbercept and aflibercept can bind placental growth factor and all isoforms of VEGF-A as well as VEGF-B (Zhang et al., 2008; Li et al., 2014; Lu and Sun, 2015; Lazzara et al., 2019) and show the advantages of multiple targets, strong affinity, and a long vitreous half-life, which is 4.2 days in rabbits, while those of aflibercept, bevacizumab, and ranibizumab are 4.79, 6.61, and 2.88 days, respectively (Lu and Sun, 2015). Moreover, brolucizumab was also approved for the treatment of nAMD in the United States recently (Markham, 2019).

Although several attempts on the efficacy and safety of conbercept have been published (Zhang et al., 2018a; Cui and Lu, 2018; Wang et al., 2018; Liu and Li, 2019; Sun et al., 2020; Wang et al., 2020), no study has pooled up-to-date data and comprehensively summarized the randomized controlled trials (RCTs) that used conbercept for the treatment of nAMD, DME, and pmCNV. Therefore, we conducted a systematic review and meta-analysis to assess whether conbercept, compared with other therapeutic regimens, is more beneficial for patients with nAMD, DME, and pmCNV in terms of efficacy, safety, compliance, and pharmacoeconomic evaluations.

## Methods

The study was conducted according to the Preferred Reporting Items for Systematic Reviews and Meta-Analyses (PRISMA) (Liberati et al., 2009) and on the statements reported in the Cochrane Handbook (Higgins et al., 2020). The PRISMA checklist and search strategies are available in Supplementary Tables S1, S2, respectively. The protocol for this review is available in PROSPERO (CRD42019147379).

### Sources and Search Methods

A systematic literature search of PubMed, Embase databases, Cochrane Library, ClinicalTrials.gov, SinoMed, China National Knowledge Infrastructure, and WanFang Data was conducted from their inception time to July 27, 2021 for RCTs and pharmacoeconomic studies of intravitreal conbercept (IVC) in nAMD, DME, and pmCNV. The language was not limited, and studies published in peer-reviewed journals or RCTs registered online with results were considered for inclusion. Hand search was also conducted on included studies and conference abstracts with available data.

RCTs and pharmacoeconomic studies were eligible if they enrolled patients with nAMD, DME, or pmCNV and compared IVC with placebo, other intravitreal anti-VEGF treatments, or conservative physical therapies. The main outcomes included visual improvement rate, mean change in visual acuity (VA), or best corrected VA (BCVA) in Early Treatment Diabetic Retinopathy Study letters, rates of gaining more than 15 letters of BCVA or losing more than 15 letters of BCVA or VA, incremental cost-utility, cost-effectiveness ratio, or incremental quality-adjusted life years (QALYs). Additional outcomes included the mean change in central retinal thickness (CRT), mean change in central macular thickness (CMT), mean change in CNV, quality-of-life (QOL) measures, number of injections, leakage area in fluorescein angiography (FA), FA rate, retinal macular pigment density, and the rates of ocular or non-ocular adverse events (AEs) or severe AEs (SAEs). Letter, errata, and literatures without peer-review, completed data, records without full text, and duplicate literatures were excluded.

### Data Collection and Quality Assessment

Basic information and study outcomes were extracted from each study by two independent reviewers (PZ and PM). For each trial, the study type, the number of cases, diagnosis, age, sex, usage, dosing, study time, intervention, comparison, follow-up time, primary outcomes, and additional outcomes were extracted and collected by two reviewers (PZ and EW) independently in predesigned tables. Discrepancies were reviewed and decided by the third reviewer (SZ).

The methodological quality for the included RCTs was assessed following the recommendations of Cochrane collaboration (Higgins et al., 2011). The quality evaluation of pharmacoeconomic study was conducted by using the Consolidated Health Economic Evaluation Reporting Standards (CHEERS) (Husereau et al., 2013). In addition, the Grading of Recommendations, Assessment, Development, and Evaluation (GRADE) system was applied to evaluate the reliability of results (Guyatt et al., 2008). Outcomes were assessed and classified independently as high, moderate, low, and very low quality. The whole evaluation above was conducted by two independent reviewers (PZ and EW), with any disagreement resolved by the third reviewer (SZ).

### Data Synthesis and Analysis

Under the guidance of the Cochrane Handbook, we used NoteExpress 3.2 software and Microsoft Excel 2016 to manage the literatures and RevMan 5.3 to perform statistical analyses and quality assessments. Data analyses were performed for different indications, treatments in control groups, and follow-up time frames. Subgroup and sensitivity analysis were performed when high heterogeneity or high risk of bias existed.

Results for dichotomous outcomes were expressed as risk ratios (RRs) with 95% confidence intervals (Cls). For continuous outcomes, we estimated the mean difference (MD) and 95% CIs. The χ^2^ test was used to compare cases. Data were considered homogeneous if I^2^ < 50% and *p* ≥ 0.1 and were analyzed using the fixed-effect model, whereas heterogeneous data were analyzed using the random-effect model. We compared the study populations, interventions, and methods of individual trials to assess clinical and methodological heterogeneity. We only carried out descriptive analyses for one trial and more than two trials with unexplainable high heterogeneity (I^2^ ≥ 50%, *p* < 0.1). A funnel plot was used to evaluate publication bias if possible. *p* < 0.05 was considered statistically significant for all analyses.

## Results

### Study Selection

**FIGURE 1:**
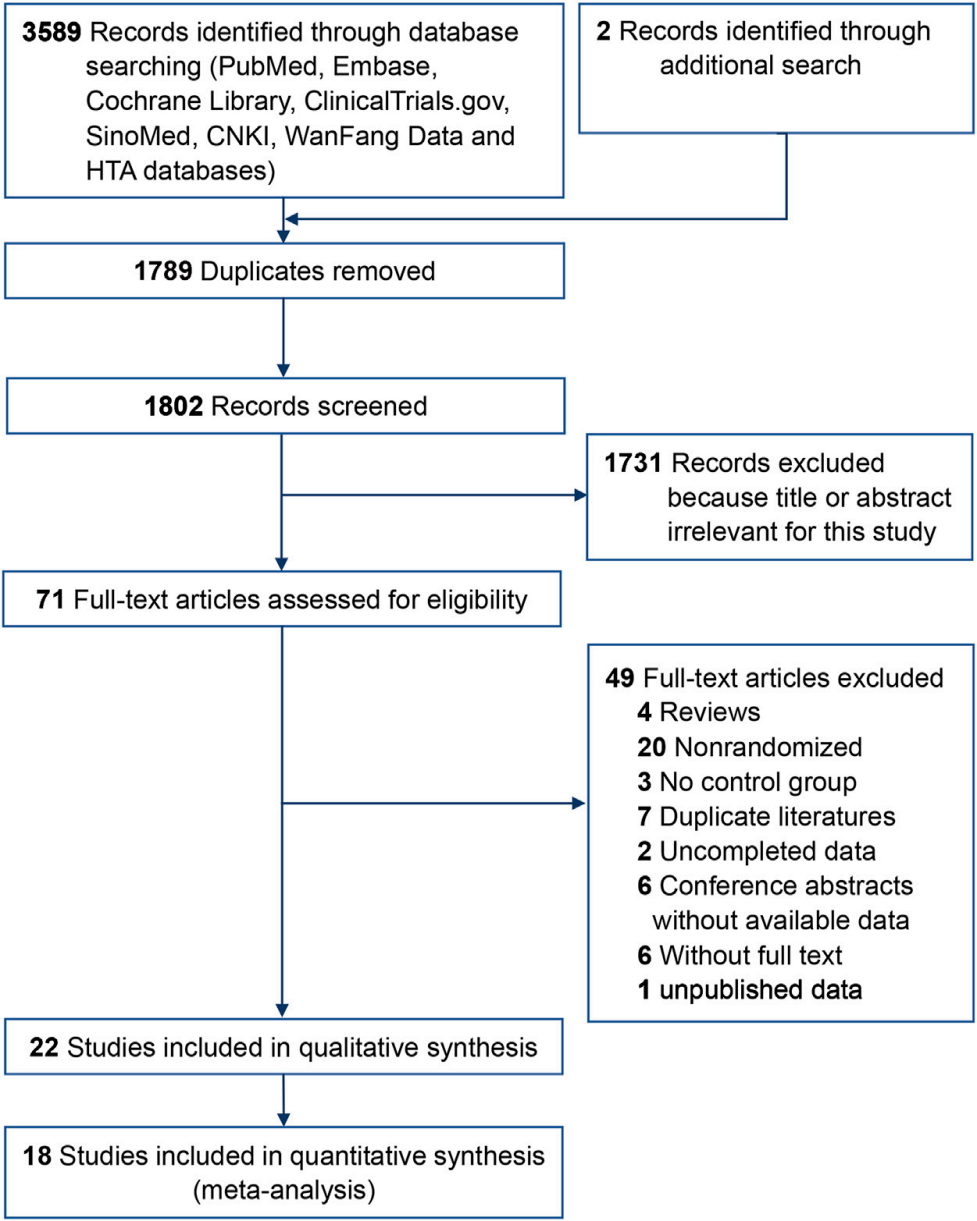
Study selection flowchart.

A total of 3,591 articles were screened for this review, while 1,789 duplicate records and another 1,802 after reviewing the titles and abstracts were excluded. Full texts of 71 records were identified and 22 trials (He et al., 2015; Liu et al., 2015; Dai et al., 2016; Du et al., 2016; Song et al., 2016; Qin et al., 2016; Han et al., 2017; Li et al., 2017; Qiao et al., 2017; Hou and Sui, 2018; Wei et al., 2018; Zhou et al., 2018; Zhang et al., 2018b; Zhang et al., 2018c; Liu et al., 2019a; Ma et al., 2019; Meng et al., 2019; Nulahou et al., 2019; Bai et al., 2020; Zhang et al., 2019a; Liu et al., 2021) (1,910 eyes) met the inclusion criteria (Figure 1), including 13 trials (He et al., 2015; Liu et al., 2015; Qin et al., 2016; Song et al., 2016; Han et al., 2017; Zhang et al., 2018b; Zhang et al., 2018c; Hou and Sui, 2018; Wei et al., 2018; Zhou et al., 2018; Liu et al., 2019a; Ma et al., 2019; Bai et al., 2020) (1,080 eyes) on nAMD and nine trials (Dai et al., 2016; Du et al., 2016; Li et al., 2017; Qiao et al., 2017; Liu et al., 2019b; Meng et al., 2019; Nulahou et al., 2019; Zhang et al., 2019b; Liu et al., 2021) (830 eyes) on DME (Table 1). Additionally, we included three pharmacoeconomic studies (Ma and Zhang, 2018; Zhang et al., 2019a; Chen and Wu, 2020).

**TABLE 1:** Characteristics of the included trials and participants.

Included trials	No. of eyes (T/C)	Age, y	Women, No. (%)	Treatment duration	Follow-up time	Treatments	Primary outcomes	Additional outcomes
**nAMD**								
Bai et al. (2020)	55/55	60∼78	52.7	NA	1 week, 1 month, and 3 months	T: IVC 0.5 mg once	VA	CRT, FA rate
						C: IVR 0.5 mg once		
Liu et al. (2019a)	81/43	58∼74	32.3	12 months	3 months, 12 months	T: IVC 0.5 mg monthly in first 3 months, and then once quarterly until 12 months	BCVA, gain or loss EDTRs letters rate	CRT, leakage area on FA, Safety
						C: sham injection monthly in first 3 months, and then 3 monthly followed by quarterly use of IVC 0.5 mg until 12 months		
Liu et al. (2015)	30/30	53∼83	NA	3 months	3 months	T: IVC 1.5 mg monthly	VA	CMT, CNV, FA rate
						C: IVR 0.5 mg monthly		
Ma et al. (2019)	45/45	60∼69	50.0	NA	1 month	T: IVC 1.0 mg once	VA	CRT, Safety, Compliance
						C: IVT 4.0 mg once		
Zhang et al. (2018b)	34/34	70.45	52.6	3 months	1 month, 3 months, and 6 months	T: IVC 0.5 mg monthly	VA	CRT, CNV, Safety
						C: IVR 0.5 mg monthly		
Hou and Sui (2018)	30/30	61∼79	40	NA	1 week, 6 months, 12 months	T: IVC 1.0 mg monthly	VA	CRT, Retinal macular pigment density
						C: IVT 4.0 mg monthly		
Zhang et al. (2018c)	38/37	58∼70	NA	NA	1 month, 3 months, and 6 months	T: IVC 0.5 mg monthly	BCVA	CRT, Safety
						C: IVT 20 mg monthly		
Song et al. (2016)	56/56	53∼82	47.3	3 months	1 month, 3 months, and 6 months	T: IVC 0.5 mg monthly	VA	CRT, Safety
						C: Conservative treatment monthly		
He et al. (2015)	30/30	55∼72	55	NA	1 day, 1 month, 3 months	T: IVC 1.0 mg once	BCVA	CRT, Safety
						C: IVT 4.0 mg once		
Han et al. (2017)	42/38	55∼87	57.1	3 months	3 months	T: IVC 0.5 mg monthly	VA improvement rate, BCVA	CRT, Safety
						C: IVT 4 mg monthly		
Wei et al. (2018)	35/35	61∼81	47.1	3 months	3 days	T: IVC 1.5 mg monthly	VA	Safety
						C: IVR 0.5 mg monthly		
Qin et al. (2016)	41/41	51∼81	48.8	3 months	1 month and 3 months	T: IVC 1.0 mg monthly	VA	CRT, CNV, FA rate
						C: Intermittent TTT		
Zhou et al. (2018)	45/44	55∼77	48.8	3 months	3 months	T: IVC 0.5 mg monthly C: IVR 1.0 mg monthly	VA improvement rate, BCVA	CRT, Safety
**DME**								
Li et al. (2017)	20/20	28∼66	47.5	3 months	1.5 months and 3 months	T: IVC 0.5 mg with LP twice in 3 months	BCVA	CMT, Safety
						C: LP once		
Dai et al. (2016)	26/26	51∼81	48.1	3 months	1 month, 3, 6, 12 months	T: IVC 1.5 mg monthly with Vitrectomy	VA	CMT, CNV
						C: Vitrectomy		
Du et al. (2016)	43/44	57∼71	46	NA	1 month, 3 months, and 6 months	T: IVC 1.0 mg +LP (1 week later) once	VA improvement rate, BCVA	CMT, Safety, Compliance
						C: LP once		
Qiao et al. (2017)	30/28	52∼73	42.5	6 months	1 month, 2 months, and 6 months	1 week to 1 month: group A: IVC 0.5 mg once; group B: IVT 40 mg twice per 2 weeks; 1.25 months–2 months: two groups exchanged	BCVA	CMT
						3 months–6 months: no intervention		
Liu et al. (2021)	125/123	50∼67	49.2	12 months	3, 6, 9, and 12 months	T: IVC, sham LP, PRN after the first injection	BCVA	CRT, number of injections
						C: LP, sham injection, PRN after the first injection		
Meng et al. (2019)	45/45	42∼80	40.0	NA	0.25 months, 0.5 months, and 1 month	T: IVC 0.5 mg + LP (1 week later) once	BCVA	CMT, Safety
						C: LP once		
Zhang et al. (2019b)	50/50	Mean: 52.76	46.0	NA	Postoperative	T: IVC 0.5 mg once with Vitrectomy	BCVA	CMT
						C: Vitrectomy		
Nulahou et al. (2019)	46/46	42∼47	42.5	NA	After injection, 3 months	T: IVC 0.5 mg + LP (1 week later) once	VA improvement rate, BCVA	CMT
						C: LP once		
Liu et al. (2019b)	32/31	55∼68	48.2	NA	1 month, 3 months, and 6 months	T: IVC 0.5 mg + LP (1 week later) once	BCVA	CMT
						C: LP once		

All studies in this table were RCTs. All of included trials were conducted in People’s Republic of China.

**Abbreviations:** T: treatment group; C: comparison group; IVC: intravitreal conbercept, IVR: intravitreal ranibizumab; IVT: intravitreal triamcinolone acetonide; LP: laser photocoagulation; NA: not available; TTT: transpupillary thermotherapy; PRN: pro re nata. VA: visual acuity; BCVA: best corrected visual acuity; CRT: central retinal thickness; CMT: center macular thickness; CNV: choroidal neovascularization; FA: fluorescein angiography.

### Quality Assessment and Risk of Bias

The risk of bias assessment results were shown in Supplementary Figure S1 and Supplementary Table S3. Twelve trials (He et al., 2015; Du et al., 2016; Song et al., 2016; Qiao et al., 2017; Zhang et al., 2018c; Wei et al., 2018; Liu et al., 2019a; Ma et al., 2019; Meng et al., 2019; Nulahou et al., 2019; Zhang et al., 2019b; Bai et al., 2020; Liu et al., 2021) presented a low risk with respect to sequence generation, whereas the risk was unclear in nine studies (Liu et al., 2015; Dai et al., 2016; Qin et al., 2016; Han et al., 2017; Li et al., 2017; Zhang et al., 2018b; Hou and Sui, 2018; Zhou et al., 2018; Liu et al., 2019b). Two trials (Liu et al., 2019a; Liu et al., 2021) presented a low risk in allocation concealment. Although the majority of information about blinding was unclear, for the three trials with low risk of bias, intravitreal injection might not cause considerable bias when the administration was the same. Seven trials (Dai et al., 2016; Du et al., 2016; Qin et al., 2016; Song et al., 2016; Li et al., 2017; Qiao et al., 2017; Meng et al., 2019) were judged as unclear risk in blinding because the records of AEs were subjective. Attrition bias was assessed as high risk in one trial with no reported reasons of withdrawal (Zhang et al., 2018c). No additional significant bias was found.

Two pharmacoeconomic studies were assessed by CHEERS with the score of 20.5 (Ma and Zhang, 2018) (high quality) and 23.5 (Chen and Wu, 2020) (high quality), but the checklist was unavailable for the included pharmacoeconomic conference abstract (Zhang et al., 2019a). Supplementary Table S5 summarized the GRADE evaluation results.

### nAMD

#### VA Outcomes

The rate of VA improvement was defined as the proportion of eyes gaining more than one line to total eyes, in accordance with the Snellen visual chart. Two trials (Han et al., 2017; Zhou et al., 2018) reported significantly higher rates of VA improvement in the IVC group (100.0 and 95.0%) than those in the intravitreal ranibizumab (IVR) group (97.8%, *p* < 0.05) or intravitreal triamcinolone acetonide (IVT) group (79.0%, *p* = 0.041).

Liu et al. (2019a) reported that the proportion of participants who lost less than 15 (100.0%) or 5 letters (93.0%) in the IVC group was higher than that in the sham injection group (93.0%, *p* = 0.02; 76.7%, *p* = 0.006) at 3 months. The proportion of patients who gained more than 10 (49.4%) or 15 letters (23.5%) in the IVC group was higher than that in the sham injection group (18.6%, *p* < 0.001; 16.3%, *p* = 0.34, respectively) at 3 months.

Liu et al. (2019a) reported a higher mean change of BCVA from baseline in the IVC group than that in the sham injection group (MD, 7.27 letters, 95% CI: 3.36 to 11.18, and *p* < 0.001) at 3 months (moderate quality). However, similar results (MD, 1.24 letters, 95% CI: −4.01 to 6.50,and *p* = 0.64) were observed in the mentioned two groups at 12 months (moderate quality) because patients in the sham injection group also received IVC treatment to prevent VA from decreasing after 3 months considering ethical requirements.

Two studies (Qin et al., 2016; Song et al., 2016) reported the changes in VA after IVC compared with physical therapies based on unaided VA or logarithm of the minimum angle of resolution (logMAR). At 1, 3, and 6 months of the treatment, changes in the VA of the IVC group were significantly higher than those of the physical therapy group (*p* < 0.05).

Five studies (Liu et al., 2015; Zhang et al., 2018c; Wei et al., 2018; Zhou et al., 2018; Bai et al., 2020) reported changes in BCVA or unaided VA after IVC treatment compared with IVR treatment. Three studies showed that BCVA scores (Zhou et al., 2018) and unaided VA improvement (Zhang et al., 2018c; Bai et al., 2020) in the IVC and IVR groups were higher than those before treatment, but the difference between the two groups was not statistically significant. Two studies (Liu et al., 2015; Wei et al., 2018) showed that at 3 days and 3 months of treatment, the VA (logMAR) of the IVC group was significantly lower than that of the IVR group (*p* < 0.05), suggesting greater effects of IVC on vision improvement.

Five studies (He et al., 2015; Han et al., 2017; Hou and Sui, 2018; Zhang et al., 2018b; Ma et al., 2019) reported the changes in BCVA and unaided VA after using IVC compared with IVT treatment. Three out of four studies (He et al., 2015; Han et al., 2017; Zhang et al., 2018b) reported changes in BCVA. Given that the high dose of IVT (20 mg) in one trial (Zhang et al., 2018b) might cause heterogeneity (I^2^ = 89%), the meta-analysis showed greater improvement of BCVA in the IVC group compared with the IVT group (1 month: MD, 0.16 letters; 95% CI: 0.13 to 0.19; and *p* < 0.00001, moderate quality; 3 months: MD, 0.11 letters; 95% CI: 0.08 to 0.20; and *p* < 0.00001,moderate quality) (Supplementary Figure S2). Two studies (Hou and Sui, 2018; Ma et al., 2019) reported that at 1, 6, and 12 months the unaided VA in the IVC group was significantly better than that in the IVT group (*p* < 0.05).

### Quantitative Measures

Twelve studies (He et al., 2015; Liu et al., 2015; Qin et al., 2016; Song et al., 2016; Han et al., 2017; Zhang et al., 2018b; Zhang et al., 2018c; Hou and Sui, 2018; Zhou et al., 2018; Liu et al., 2019a; Ma et al., 2019; Bai et al., 2020) reported the changes in quantitative measures before and after the treatment, including CRT, CMT, CNV, and FA.

Liu et al. (2019a) reported that the changes in the CRT of the IVC group were significantly less than that in the sham injection group at 3 months (MD, −83.29 μm; 95% CI, −125.92 to −40.67; and *p* < 0.001, low quality), whereas no significant difference was found between the two groups at 12 months (MD, 4.08 μm, 95% CI: −19.75 to 47.90, *p* = 0.41, and low quality).

The meta-analysis of two studies (Qin et al., 2016; Song et al., 2016) showed that the CRT change in the IVC group was significantly smaller (MD, −18.90 μm; 95% CI: −37.08 to −0.73; and *p* = 0.04, low quality) at 3 months than that in the conservative treatment group (Supplementary Figure S3). Qin et al. (2016) also reported that the CNV area and the leakage area on FA in the IVC group were significantly lower than those in the conservative treatment group, indicating the better visual improvement effect of IVC from the anatomical point of view.

Four studies (Liu et al., 2015; Zhang et al., 2018c; Zhou et al., 2018; Bai et al., 2020) reported the difference in the improvement of CRT in the IVC group compared with that in the IVR group. However, the dosage and number of IVR injections in three trials (Zhang et al., 2018c; Zhou et al., 2018; Bai et al., 2020) may result in a high heterogeneity (I^2^ = 98%), leading to only one conducted descriptive analysis. All patients in the three trials had smaller CRT after treatment, whereas two trials (Zhou et al., 2018; Bai et al., 2020) showed that the IVC group had smaller CRT than the IVR group (*p* < 0.05). However, Zhang et al. (2018c) showed a larger CRT in the IVC group than the IVR group, but no statistically significant difference in both groups after 3 months of treatment, with a high risk of bias in selective reporting.

Pooled results of two studies (Liu et al., 2015; Zhang et al., 2018c) showed that the CNV area in the IVC group was significantly smaller than that in the IVR group (MD, −1.26 mm^2^; 95% CI: −1.59 to −0.93; *p* < 0.00001, very low quality) at 3 months (Supplementary Figure S4). Liu et al. (2015) reported the changes in CMT and FA rate. It showed that at 3 months, the CMT and FA rate in the IVC group were significantly smaller than those in the IVR group. Hou and Sui (2018) showed a significant improvement in the macular pigment density of the IVC group (6 months: 0.23DU ± 0.05, 12 months: 0.27DU ± 0.06) compared with that in the IVR group (6 months: 0.13DU ± 0.04 and 12 months: 0.14DU ± 0.05).

Meta and sensitivity analyses of four studies (He et al., 2015; Han et al., 2017; Zhang et al., 2018b; Ma et al., 2019) showed that the CRT change in the IVC group was larger at 1 month (MD, −89.35 μm; 95% CI: −102.71 to −75.99; *p* < 0.00001, low quality) and 3 months (MD, −102.99 μm; 95% CI: −131.60 to −74.37; *p* < 0.00001, low quality) than that in the IVT group (Supplementary Figure S5). Two trials (Zhang et al., 2018b; Hou and Sui, 2018) showed that the patients in the IVC group had larger CRT reduction than those in the IVT group at 6 months.

### Safety

Nine studies (He et al., 2015; Song et al., 2016; Han et al., 2017; Zhang et al., 2018b; Zhang et al., 2018c; Wei et al., 2018; Zhou et al., 2018; Liu et al., 2019a; Ma et al., 2019) reported the AEs related to IVC in the treatment of nAMD.

Liu et al. (2019a) reported that IVC was well tolerated. At 3 months, the incidence rates of ocular and non-ocular AEs in the IVC group were both 28.4%, whereas the incidence rates in the sham injection group were 18.6% (*p* = 0.23) and 25.6% (*p* = 0.74), respectively. Song et al. (2016) reported the safety of IVC compared with conservative therapies and showed temporary subconjunctival hemorrhage (26.8%) and ocular hypertension (17.9%) in the IVC group, whose patients recovered in a short period of time.

Three (Zhang et al., 2018c; Wei et al., 2018; Zhou et al., 2018) out of four studies (He et al., 2015; Han et al., 2017; Zhang et al., 2018b; Ma et al., 2019) reported the safety of the IVC group compared with the IVR group (low quality) and the IVC group compared with the IVT group (moderate quality), respectively. Meta-analyses showed no significant difference in the incidence of subconjunctival hemorrhage and ocular hypertension in the two comparisons (Supplementary Figures S6, S7, respectively). No ocular SAE was reported in both groups.

### DME

#### VA Outcomes

Two studies (Du et al., 2016; Nulahou et al., 2019) reported that the VA improvement rate was significantly higher in the IVC with the LP group compared with that in the LP alone group at 3 (91.30%; 69.57%, *p* = 0.009) (Nulahou et al., 2019) and 6 months (81.40%; 13.64%, *p* < 0.05) (Du et al., 2016), respectively.

Meta-analyses of five trials (Du et al., 2016; Li et al., 2017; Meng et al., 2019; Nulahou et al., 2019; Liu et al., 2019b) showed that the improvement of BCVA in the IVC with the LP group was larger than that in the LP alone group at 1 (MD, 0.04 letters; 95% CI: 0.03 to 0.06; and *p* < 0.00001, moderate quality) and 3 months (MD, 0.09 letters; 95% CI: 0.07 to 0.11; and *p* < 0.00001, moderate quality) after excluding the study from Liu et al. (2019b) in the sensitivity analysis (from I^2^ = 63% to I^2^ = 0%) (Supplementary Figure S8). After 6 months of treatment, the improvement of BCVA was better in the IVC with the LP group than that in the LP alone group in one study (Du et al., 2016), whereas no significant difference was observed between the two groups in the other study (Liu et al., 2019b). A trial (Liu et al., 2021) reported that BCVA improvement was better in the IVC monotherapy group from 1 month to 12 months (8.21 letters ± 9.50 vs. 0.26 letters ± 12.00) compared with the LP therapy.

Two trials reported that compared with the VT group, the IVC with the VT group presented a significantly better BCVA improvement after treatment of 3 months (*p* < 0.001) (Zhang et al., 2019b) and 1 year (*p* < 0.05) (Dai et al., 2016). Additionally, Qiao (Qiao et al., 2017) identified better improvement at 1 week (*p* = 0.03) in the IVC group compared with the IVT group, whereas the difference was not significant at 1 month (*p* = 0.07).

### Quantitative Measures

Five trials (Du et al., 2016; Li et al., 2017; Liu et al., 2019b; Meng et al., 2019; Nulahou et al., 2019) reported that the IVC with the LP group presented lower CMT values at 1 month (MD, −15.16 μm; 95% CI: −25.31 to −5.02; and *p* = 0.003; low quality) and 3 months (MD, −24.51 μm; 95% CI: −35.55 to −13.46;and *p* < 0.0001, low quality) than the LP group (Supplementary Figure S9). At 6 months, two trials were included with a high heterogeneity. Thus, the descriptive analysis was performed. Du et al. (2016) reported that the IVC with the LP group had lower CMT values than the LP alone group. However, Liu et al. (2019b) showed no difference in the two groups. In addition, two trials (Dai et al., 2016; Zhang et al., 2019a) reported that the IVC with the VT group had low CMT values at 1 and 3 months (*p* < 0.05). Compared with the LP group (−130 µm ± 190), the IVC group (−200 µm ± 210) showed lower CRT values at 1 year (Liu et al., 2021). Qiao et al. (2017) reported that the CMT values were lower in the IVC group than those in the IVT group at 1 week (*p* = 0.003) and 1 month (*p* = 0.02), but no statistical differences were observed at 1.25 and 6 months.

### Safety

Three studies (Du et al., 2016; Li et al., 2017; Meng et al., 2019) reported the AEs related to IVC in the treatment of DME. Subconjunctival hemorrhage (Li et al., 2017) and ocular hypertension (Du et al., 2016; Li et al., 2017; Meng et al., 2019) were reported as AEs, and no significant difference was observed in the IVC and control groups. All patients with AEs recovered after symptomatic treatment and no SAE occurred.

### Compliance and Pharmacoeconomic Evaluation

#### Compliance

Two trials (Du et al., 2016; Ma et al., 2019) reported the compliance to the use of IVC by the QOL questionnaire, and the results demonstrated that higher QOL scores in self-care ability, activity ability, interpersonal relationship, psychology, and physiology domains were achieved in patients with IVC treatment compared with those under IVR in nAMD (Ma et al., 2019) or LP in DME (Du et al., 2016).

#### Pharmacoeconomic Evaluation

Ma and Zhang (2018) used the Markov model to simulate the outcomes, utility, and cost of nAMD under IVC and IVR treatments. The clinical, output, and cost data were obtained in the AURORA (Li et al., 2014) and ANCHOR (Zhang et al., 2019b) trials, respectively. And the pharmacoeconomic evaluation was carried out without head-to-head data by the cost–utility analysis. The IVR group spent ¥187,692.72 more than the IVC group for each additional QALY, and this value was over three times the threshold of GDP per capita (¥150,753) in China in 2016, with a discount rate of 3%. In addition, univariate and probabilistic sensitivity analyses (PSA) demonstrated the robustness of the results. Therefore, IVC was more economical for the treatment of nAMD compared with IVR in China.

With the publication of the latest PHOENIX trial (Liu et al., 2019a), Chen and Wu (2020) assessed the cost-effectiveness of IVC in the treatment of nAMD compared with other anti-VEGF therapies using the Markov model in a Chinese healthcare setting. IVC produced smaller incremental cost-effectiveness ratios ($19,028/QALY) than IVR ($29,857/QALY q4; $20,338/QALY PRN) or intravitreal aflibercept (IVA) ($28,892/QALY) in 2018 ($1 = ¥6.8) with a discount rate of 5%. The cost per QALY of IVC against conservative treatment with a lifetime horizon was $25,849, which was below three times the threshold of gross domestic product per capita ($28,410) in 2018, whereas other anti-VEGF regimens did not present as cost-effectiveness alternatives. PSA also indicated that IVC performed greatest probabilities of cost-effectiveness (92%) compared with other strategies.

Zhang et al. (2019a) conducted a cost-effectiveness analysis with a Markov model of IVC versus IVR in the treatment of pmCNV from the perspective of Chinese payers. IVC and IVR had similar total costs and QALYs (¥189,207 versus ¥175,955; 9.86 versus 9.83), whereas PSA results demonstrated that IVC had a slightly high level of cost-effectiveness in China.

## Discussion

This meta-analysis was designed to comprehensively evaluate the efficacy, safety, economy, and compliance of IVC, a new VEGF regimen, in the treatment of nAMD and visual impairment due to DME or pmCNV. To the best of our knowledge, this is the first systematic review and meta-analysis that contains all three approved indications without limitation of comparisons, with up-to-date studies and GRADE evaluation. Therefore, the conclusion of this review could be credible and comprehensive, providing the latest update on the systematic review of IVC.

In this review, we found that patients with nAMD or visual impairment due to DME receiving IVC achieved better improvement in VA and quantitative measures than those under placebo, IVT, LP treatments, and gained comparable efficacy to IVR. This may potentially be explained by conbercept’s unique pharmacodynamic characteristics. Intravitreal anti-VEGF agents inhibit the functional activity of proangiogenic factors with different target selectivity, affinity, and potency (Fogli et al., 2018). Conbercept exhibits a higher affinity to VEGF (Kd = 0.5 pM) than ranibizumab (Kd = 46 pM) and bevacizumab (Kd = 58 pM), but is similar to aflibercept (Kd = 0.5 pM) (Lu and Sun, 2015), because of the addition of the fourth Ig-like domain of VEGFR-2 in the Fab fragment (Zhang et al., 2009). Nevertheless, partial results suggested that long-term IVC treatment may have decreased effectiveness in vision improvement, but this speculation needs to be confirmed in long-term investigations. Additionally, the number of injections should also be investigated among anti-VEGF agents. Fewer injections can lead to better efficacy as PRN treatment, lower risk of AEs, preferable compliance, and lower cost.

So far, several therapeutic schedules have been developed for retinal diseases. LP has taken a back seat to therapies in the treatment of macular diseases, owing to the unsatisfactory vision gain, complications, and AEs, such as subretinal fibrosis and laser scars (Lock and Fong, 2011; Chhablani et al., 2018). ITV, a steroidal drug, has been widely used for retinal conditions. However, due to its complications, such as secondary ocular hypertension (20–40%), steroid-induced cataract (15–20%), and endophthalmitis (<1%), the optimal balance between efficacy and safety profile of steroid use has yet to be completely determined (Veritti et al., 2012). As a result, anti-VEGF agents have been widely used as first-line treatments of various angiogenesis-driven eye diseases. To date, published Cochrane network meta-analyses involving more than 10,000 participants have demonstrated that anti-VEGF agents, except IVC, are comparably effective in terms of maintaining VA, while the effects of long-term use and safety of anti-VEGF agents needs further investigation (Virgili et al., 2018; Solomon et al., 2019).

Close monitoring is needed in the immediate and subsequent periods post drug administration, although this review indicated that IVC might not increase the risk of ocular or potential systematic AEs compared with other treatments. Intravitreal anti-VEGF agents may be associated with devastating complications, such as endophthalmitis, intraocular inflammation, rhegmatogenous retinal detachment, intraocular pressure elevation, ocular haemorrhage, etc. (Falavarjani and Nguyen, 2013), but do not increase the risk of systematic AEs (Thulliez et al., 2018). Except for the surgical technique by ophthalmologists and the aseptic preparation of syringes (Scott and Flynn, 2007), various factors, including the suppression of VEGF levels, number of injections (Hoang et al., 2012), or even anticoagulant therapy (Mason et al., 2010), can be related to increased risk of AEs. Therefore, routine monitoring and detailed records of ocular or non-ocular complications are recommended in all patients receiving IVC.

Only a few studies assessed QOL and compared the experience of receiving IVC therapy with other anti-VEGF agents from a patient perspective, and this review suggested that patients on IVC therapy may more likely adhere to treatment satisfactorily. While anti-VEGF injections represent the mainstay of current treatment for retinal diseases, attention should be paid to patient compliance, which can be affected by multiple factors. Surgical factors have been shown to influence pain and discomfort related to the injection procedure, including the number of injections, instillation of eye drops, use of surgical drapes, and needle entry (Boyle et al., 2015). Demographical and psychological factors were associated with compliance, such as advanced age, unfavorable change in VA, fear of ophthalmic surgery, vision loss, AEs, and other unknown situations (Senra et al., 2016; Ehlken et al., 2020). Moreover, social factors, such as financial burden of drugs, nursing and follow-up, and distance to the treatment center, also played a vital role in the preference to ocular injection (Boyle et al., 2018; Ehlken et al., 2020). Therefore, further assessments of patients’ compliance to intravitreal anti-VEGF agents should be accomplished in future studies periodically and at the end of the study.

Retinal diseases and the increasing need for anti-VEGF therapy are emerging as a global health issue and economic burden affecting both developing and developed countries (Hodgson et al., 2016). However, different prices and accessibility of anti-VEGF regimens, indirect costs, and healthcare system expenditures among countries and regions (Parikh et al., 2019) can lead to different medication choices. In the past few years, there has been an increasing use of IVA and IVR worldwide, while intravitreal bevacizumab (IVB), with comparable effectiveness, safety, and cost-saving advantage, occupies the main position in the United States from 2006 to 2015 (Parikh et al., 2017). As regards the off-label use of IVB in ophthalmology in Chinese clinical practice, no trial has performed head-to-head comparison of IVC with IVB. We reported that IVC has a cost–utility advantage over IVR and cost-effective advantage over IVA, IVR, and conservative care in the treatment of nAMD and pmCNV in China. However, compared to high-income regions, the pricing negotiation policy in the Chinese healthcare system have led to the reduction of anti-VEGF regimens prices and also caused small differences between them in recent years (Li et al., 2018). Therefore, the prices of anti-VEGF regimens may be the most important factor affecting the economic benefits as the efficacy and safety of anti-VEGF regimens tends to be comparative. Further studies are needed to determine whether IVC has economic advantages when treating DME in China and other conditions worldwide.

Although several systematic reviews of IVC have been published (Zhang et al., 2018a; Cui and Lu, 2018; Wang et al., 2018; Liu and Li, 2019; Sun et al., 2020; Wang et al., 2020), their limitations and biases were considered uncontrollable, which could result in unreliability of the conclusions. Thus, all studies included in these reviews had been rechecked thoroughly (Supplementary Table S6). Except for the trials finally included in this analysis, the remaining studies were considered to be nonrandomized clinical trials, retrospective studies, or had inappropriate comparisons and were thus excluded in this review.

### Limitations

First of all, the included trials only enrolled Chinese patients, which might lead to reporting bias in ethnicity. Although the advances in intravitreal injection were all completed by experienced ophthalmologists, clinical heterogeneity can be carried out across included studies. The selection bias in most of the included trials was characterized by unclear risk, indicating that several subjective outcomes, such as compliance evaluation and judgment of AEs, could be influenced consequently.

In addition, the sample size in selected studies may be insufficient for measuring the primary and secondary endpoints, resulting in downgrading in the GRADE evaluation. We did not include the unpublished data in pmCNV on clinicaltrials.gov (Nct., 2013), which was not peer-reviewed and could yield inevitable uncertainty and bias to the conclusion. In the absence of head-to-head trials between IVC and IVA, the superiority of IVC compared with different approved anti-VEGF agents should be further investigated by performing network meta-analyses.

## Conclusion

In summary, this systematic review and meta-analysis suggests that IVC is well tolerated and effective for improving vision acuity and quantitative measures in the fundus condition in patients with nAMD and DME comparing with LP, IVT, and placebo, and gains comparable efficacy to IVR. However, well-designed, large-sample and long-term evaluation of IVC shall be conducted in additional studies worldwide.

## Abbreviations

AMD, age-related macular degeneration; nAMD, neovascular age-related macular degeneration; DME, diabetic macular edema; pmCNV, pathologic myopia choroidal neovascularization; VEGF, vascular endothelial growth factor; LP, laser photocoagulation; RCTs, randomized controlled trials; PRISMA, preferred reporting items for systematic reviews and meta-analyses; IVC, intravitreal conbercept; VA, visual acuity; BCVA, best corrected visual acuity; QALYs, quality-adjusted life years; CRT, central retinal thickness; CMT, mean change in central macular thickness; QOL, quality-of-life; FA, fluorescein angiography; AEs, adverse events; SAEs, severe adverse events; CHEERS, consolidated health economic evaluation reporting standards; GRADE, grading of recommendations assessment, development and evaluation; RRs, risk ratios; CIs, confidence intervals; MD, mean difference; IVR, intravitreal ranibizumab; IVT, intravitreal triamcinolone; logMAR, logarithm of minimum angle resolution; PSA, probabilistic sensitivity analyses; PRN, pro re nata; IVA, intravitreal aflibercept; IVB, intravitreal bevacizumab.
