# Supplementary material for: Conbercept for Treatment of Neovascular Age-Related Macular Degeneration and Visual Impairment due to Diabetic Macular Edema or Pathologic Myopia Choroidal Neovascularization: A Systematic Review and Meta-Analysis
Source: Front Pharmacol. 2021 Oct 12;12:696201. doi: 10.3389/fphar.2021.696201 (PMC8546330; doi:10.3389/fphar.2021.696201)
Supplement: Supplementary file 1 [file DataSheet1.docx]

**Supplementary Materials**

**eFigure 1 Risk of Bias assessment**

**
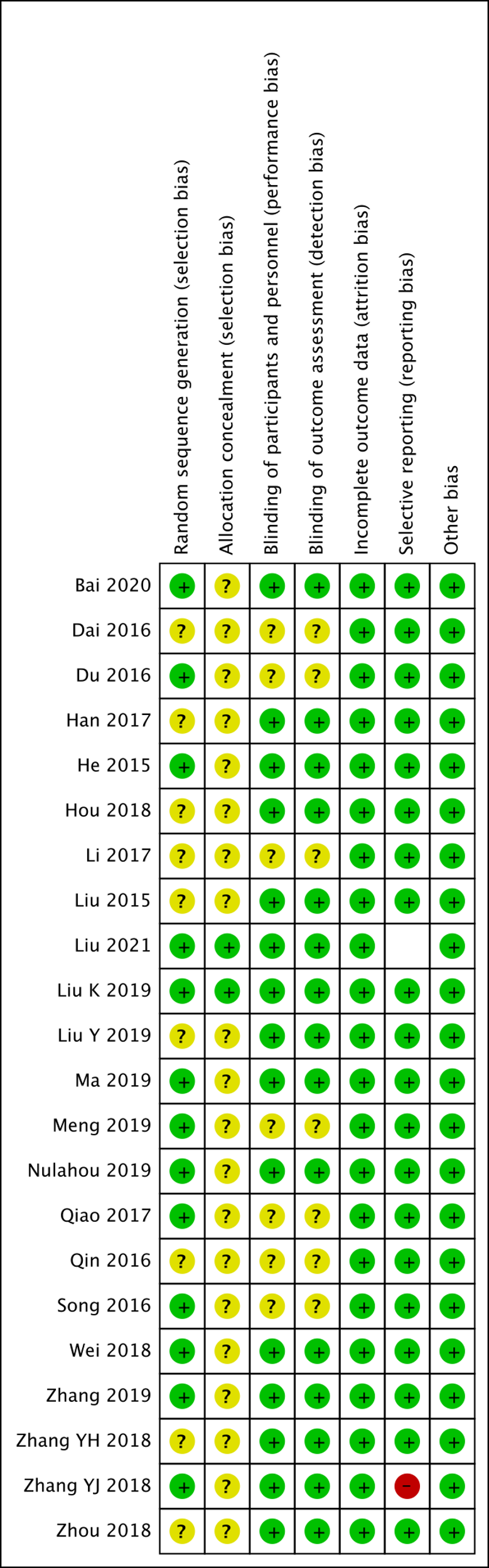
**

**eFigure 2 Forest Plot of BCVA values obtained after 1-month treatment and 3-month treatment of IVC compared with IVT in nAMD**


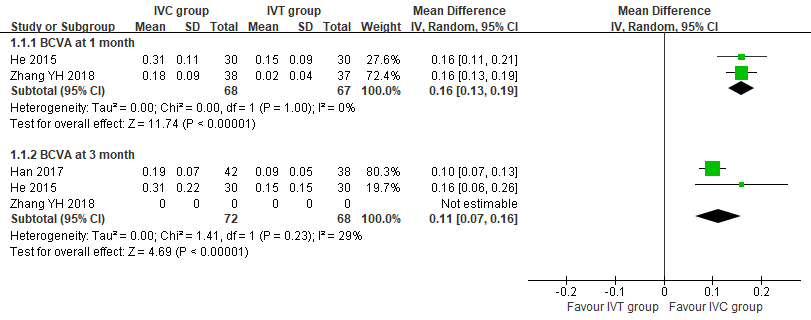


**eFigure 3 Forest Plot of CRT values obtained after 3-month treatment of IVC compared with conservative treatment in nAMD**


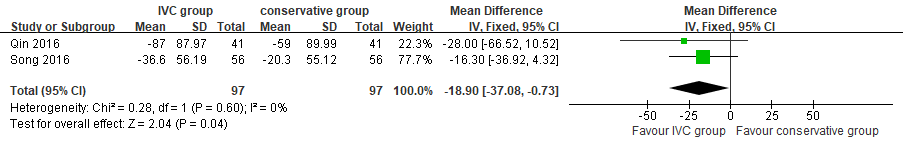


**eFigure 4 Forest Plot of CNV area values obtained after 3-month treatment of IVC compared with IVR in nAMD**


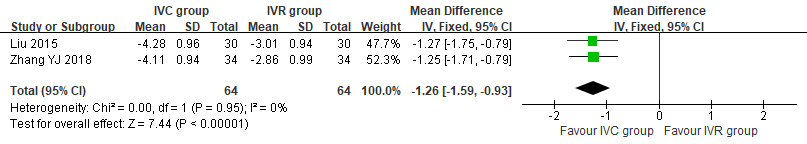


**eFigure 5 Forest Plot of CRT values obtained after 1-month treatment and 3-month treatment of IVC compared with IVT in nAMD**


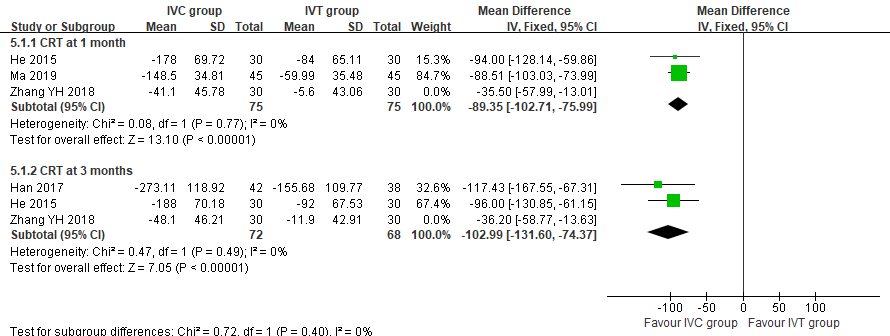


**eFigure 6 Forest Plot of the incidence of AEs after treatments of IVC compared with IVR in nAMD**


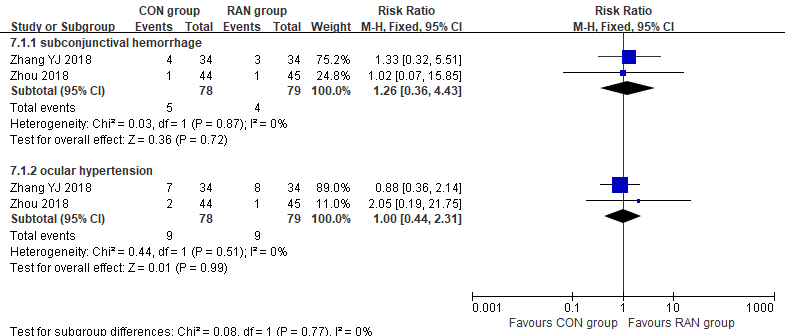


**eFigure 7 Forest Plot of the incidence of AEs after treatments of IVC compared with IVT in nAMD**

**
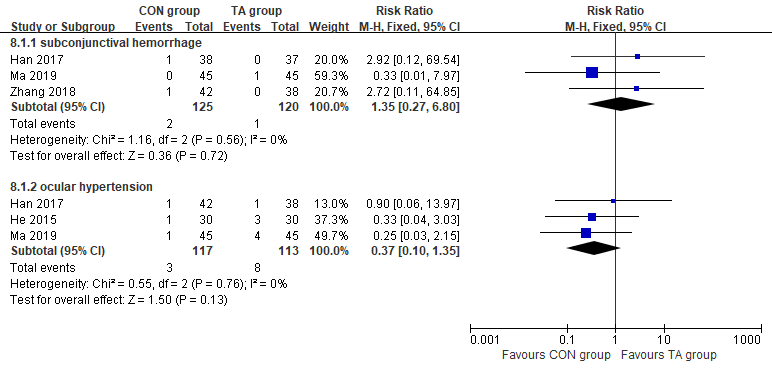
**

**eFigure 8 Forest Plot of BCVA values obtained after 1-month treatment and 3-month treatment of IVC with LP compared with LP in DME**


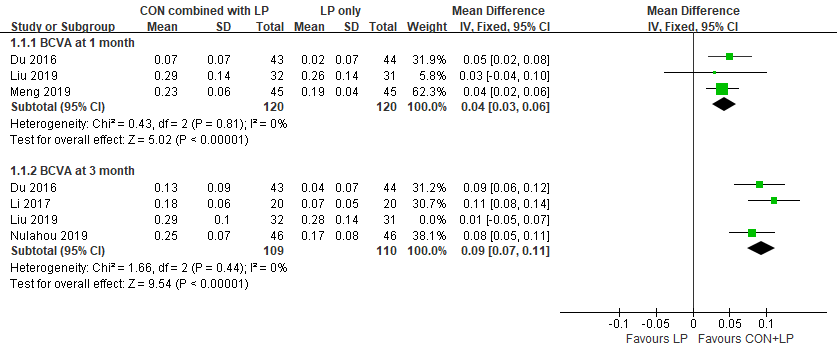


**eFigure 9 Forest Plot of CMT values obtained after 1-month treatment and 3-month treatment of IVC with LP compared with LP in DME**


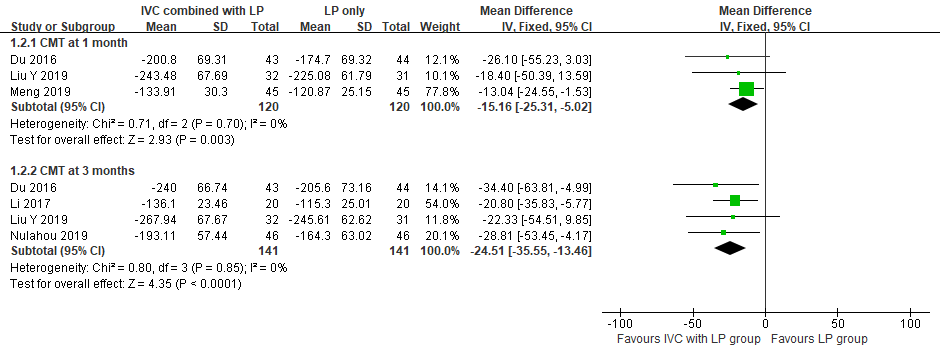


**eTable 1 Search Strategies**

| **Database** | **Search Strategies** |
| --- | --- |
| PubMed | KH902 fusion protein"[Supplementary Concept] OR "KH902 fusion protein"[All Fields] OR "conbercept"[All Fields] |
| Embase | 'conbercept'/exp OR 'conbercept' |
| The Cochrane Library | conbercept in All Text - (Word variations have been searched) |
| CNKI^*^ | Conbercept [All Fields] |
| SinoMed^*^ | Conbercept [All Fields] |
| WanFang Data^*^ | Conbercept [All Fields] |
| * Chinese Databases. The search strategies were translated to English. | |

**eTable 2 PRISMA checklist**

| **Section/topic** | **#** | **Checklist item** | **Reported on page #** |
| --- | --- | --- | --- |
| **TITLE** | | |  |
| Title | 1 | Identify the report as a systematic review, meta-analysis, or both. | 1 |
| **ABSTRACT** | | |  |
| Structured summary | 2 | Provide a structured summary including, as applicable: background; objectives; data sources; study eligibility criteria, participants, and interventions; study appraisal and synthesis methods; results; limitations; conclusions and implications of key findings; systematic review registration number. | 2-3 |
| **INTRODUCTION** | | |  |
| Rationale | 3 | Describe the rationale for the review in the context of what is already known. | 4-5 |
| Objectives | 4 | Provide an explicit statement of questions being addressed with reference to participants, interventions, comparisons, outcomes, and study design (PICOS). | 5 |
| **METHODS** | | |  |
| Protocol and registration | 5 | Indicate if a review protocol exists, if and where it can be accessed (e.g., Web address), and, if available, provide registration information including registration number. | 6 |
| Eligibility criteria | 6 | Specify study characteristics (e.g., PICOS, length of follow-up) and report characteristics (e.g., years considered, language, publication status) used as criteria for eligibility, giving rationale. | 6-7 |
| Information sources | 7 | Describe all information sources (e.g., databases with dates of coverage, contact with study authors to identify additional studies) in the search and date last searched. | 6-7 |
| Search | 8 | Present full electronic search strategy for at least one database, including any limits used, such that it could be repeated. | 6, eTable 1 |
| Study selection | 9 | State the process for selecting studies (i.e., screening, eligibility, included in systematic review, and, if applicable, included in the meta-analysis). | 7 |
| Data collection process | 10 | Describe method of data extraction from reports (e.g., piloted forms, independently, in duplicate) and any processes for obtaining and confirming data from investigators. | 7 |
| Data items | 11 | List and define all variables for which data were sought (e.g., PICOS, funding sources) and any assumptions and simplifications made. | 8 |
| Risk of bias in individual studies | 12 | Describe methods used for assessing risk of bias of individual studies (including specification of whether this was done at the study or outcome level), and how this information is to be used in any data synthesis. | 8 |
| Summary measures | 13 | State the principal summary measures (e.g., risk ratio, difference in means). | 8 |
| Synthesis of results | 14 | Describe the methods of handling data and combining results of studies, if done, including measures of consistency (e.g., I^2^) for each meta-analysis. | 8 |

| **Section/topic** | **#** | **Checklist item** | **Reported on page #** |
| --- | --- | --- | --- |
| Risk of bias across studies | 15 | Specify any assessment of risk of bias that may affect the cumulative evidence (e.g., publication bias, selective reporting within studies). | 7 |
| Additional analyses | 16 | Describe methods of additional analyses (e.g., sensitivity or subgroup analyses, meta-regression), if done, indicating which were pre-specified. | 8 |
| **RESULTS** | | |  |
| Study selection | 17 | Give numbers of studies screened, assessed for eligibility, and included in the review, with reasons for exclusions at each stage, ideally with a flow diagram. | 8-9, Figure 1 |
| Study characteristics | 18 | For each study, present characteristics for which data were extracted (e.g., study size, PICOS, follow-up period) and provide the citations. | 8-9, Table 1 |
| Risk of bias within studies | 19 | Present data on risk of bias of each study and, if available, any outcome level assessment (see item 12). | 9, eFigure 1 |
| Results of individual studies | 20 | For all outcomes considered (benefits or harms), present, for each study: (a) simple summary data for each intervention group (b) effect estimates and confidence intervals, ideally with a forest plot. | 9-17, Figure 2-9 |
| Synthesis of results | 21 | Present results of each meta-analysis done, including confidence intervals and measures of consistency. | 9-17 |
| Risk of bias across studies | 22 | Present results of any assessment of risk of bias across studies (see Item 15). | 9-17 |
| Additional analysis | 23 | Give results of additional analyses, if done (e.g., sensitivity or subgroup analyses, meta-regression [see Item 16]). | 9-17 |
| **DISCUSSION** | | |  |
| Summary of evidence | 24 | Summarize the main findings including the strength of evidence for each main outcome; consider their relevance to key groups (e.g., healthcare providers, users, and policy makers). | 17-20 |
| Limitations | 25 | Discuss limitations at study and outcome level (e.g., risk of bias), and at review-level (e.g., incomplete retrieval of identified research, reporting bias). | 20-21 |
| Conclusions | 26 | Provide a general interpretation of the results in the context of other evidence, and implications for future research. | 21 |
| **FUNDING** | | |  |
| Funding | 27 | Describe sources of funding for the systematic review and other support (e.g., supply of data); role of funders for the systematic review. | 21 |

*From:*  Moher D, Liberati A, Tetzlaff J, Altman DG, The PRISMA Group (2009). Preferred Reporting Items for Systematic Reviews and Meta-Analyses: The PRISMA Statement. PLoS Med 6(7): e1000097. doi:10.1371/journal.pmed1000097

For more information, visit: **www.prisma-statement.org**.

| eTable 3 Risk of Bias Assessment Results | | | | | | | |
| --- | --- | --- | --- | --- | --- | --- | --- |
| Included Studies | **Random Sequence generation** | **Allocation concealment** | **Blinding of participants and personnel** | **Blinding of outcomes assessment** | **Incomplete outcome data** | **Selective reporting** | **Other bias** |
| nAMD | | | | | | | |
| Liu K 2019 | L | L | L | L | L | L | L |
| Song 2016 | L | U | U | U | L | L | L |
| Qin 2016 | U | U | U | U | L | L | L |
| Zhang YJ 2018 | L | U | L | L | H | L | L |
| Zhou 2018 | U | U | L | L | L | L | L |
| Liu 2015 | U | U | L | L | L | L | L |
| Wei 2018 | L | U | L | L | L | L | L |
| Bai 2020 | L | U | L | L | L | L | L |
| Han 2017 | U | U | L | L | L | L | L |
| He 2015 | L | U | L | L | L | L | L |
| Hou 2018 | U | U | L | L | L | L | L |
| Zhang YH 2018 | U | U | L | L | L | L | L |
| Ma 2019 | L | U | L | L | L | L | L |
| DME | | | | | | | |
| Dai 2016 | U | U | U | U | L | L | L |
| Zhang 2019 | L | U | L | L | L | L | L |
| Li 2017 | U | U | U | U | L | L | L |
| Du 2016 | L | U | U | U | L | L | L |
| Nulahou 2019 | L | U | L | L | L | L | L |
| Liu Y 2019 | U | U | L | L | L | L | L |
| Meng 2019 | L | U | U | U | L | L | L |
| NCT02194634 | L | L | L | L | L | NA | L |
| Qiao 2017 | L | U | U | U | L | L | L |
| pmCNV | | | | | | | |
| NCT01809223 | L | L | L | L | L | NA | L |
| Abbreviations: U: unclear; L: Low; H: High; NA: not applicable  Random sequence generation was minimization method (NCT 01809223) and random number tables (other trials). Allocation concealment: Liu K 2019, NCT02194634 and NCT01809223 all had study center and allocation were kept in opaque containers. Ji 2018 did not report the reason of 12 dropout cases. Selective reporting was not evaluated in two unpublished trials (NCT02194634 and NCT01809223). | | | | | | | |

**eTable 4 CHEERS checklist**

| **Study** | **Item** | | | | | | | | | | | | | | | | | | | | | | | | |
| --- | --- | --- | --- | --- | --- | --- | --- | --- | --- | --- | --- | --- | --- | --- | --- | --- | --- | --- | --- | --- | --- | --- | --- | --- | --- |
|  | **1** | **2** | **3** | **4** | **5** | **6** | **7** | **8** | **9** | **10** | **11** | **12** | **13** | **14** | **15** | **16** | **17** | **18** | **19** | **20** | **21** | **22** | **23** | **24** | **Total score** |
| **AMD** | | | | | | | | | | | | | | | | | | | | | | | | | |
| Ma 2018 | 1 | 1 | 1 | 1 | 1 | 1 | 1 | 1 | 1 | 1 | 1 | 0 | 1 | 1 | 1 | 1 | 1 | 1 | 1 | 1 | 0.5 | 1 | 0 | 0 | 20.5 |
| Chen 2020 | 1 | 1 | 1 | 1 | 1 | 1 | 1 | 1 | 1 | 1 | 1 | 1 | 1 | 1 | 1 | 1 | 1 | 1 | 1 | 1 | 0.5 | 1 | 1 | 1 | 23.5 |
| Title and abstract: 1. Title; 2. Abstract; Introduction: 3. Background and objectives; Methods: 4. Target population and subgroups; 5. Setting and location; 6. Study perspective; 7. Comparators; 8. Time horizon; 9. Discount rate; 10. Choice of health outcomes; 11. Measurement of effectiveness; 12. Measurement and evaluation of preference based outcomes; 13. Estimating resources and costs; 14. Currency, price date, and conversion; 15. Choice of model; 16. Assumptions; 17. Analytical methods; Results: 18. Study parameters; 19. Incremental costs and outcomes; 20. Characterizing uncertainty; 21. Characterizing heterogeneity; Discussion: 22. Study findings, limitations, generalizability, and current knowledge; Other: 23. Source of funding; 24. Conflicts of interest | | | | | | | | | | | | | | | | | | | | | | | | | |

| **eTable 5 Summary of GRADE system evaluation results** | | | | | | | | | | | | | | | | | |
| --- | --- | --- | --- | --- | --- | --- | --- | --- | --- | --- | --- | --- | --- | --- | --- | --- | --- |
| **No. of studies, design** | | **Quality assessment** | | | | | | | | | **Summary of findings** | | | | | | |
|  |  | **Limitations** | **Inconsistency** | | **Indirectness** | | **Imprecision^#^** | **Publication bias** | | **Downgrading** | **Sample size** | | | **Treatment measures**  **95% Cl** | | **Quality** | |
|  |  |  |  |  |  |  |  |  |  |  | **Trial group** | | **Control group** |  |  |  |  |
| **nAMD** | | | | | | | | | | | | | | | | | |
| **Efficacy** | | | | | | | | | | | | | | | | | |
| **BCVA improvement at 3 months**  1 RCT | | Not serious | Not serious | | Not serious | | Serious | Undetected | | Yes | IVC | | Sham injection | MD 7.27, [3.36, 11.18] | | Moderate | |
| **BCVA improvement at 12 months**  1 RCT | | Not serious | Not serious | | Not serious | | Serious | Undetected | | Yes | IVC | | Sham injection | MD 1.24, [-4.01, 6.50] | | Moderate | |
| **BCVA improvement at 1 month**  2 RCTs | | Not serious | Not serious | | Not serious | | Serious | Undetected | | Yes | IVC, n=68 | | IVT, n=67 | RR 0.16 [0.13, 0.19] | | Moderate | |
| **BCVA improvement at 3 months**  2 RCTs | | Not serious | Not serious | | Not serious | | Serious | Undetected | | Yes | IVC, n=72 | | IVT, n=68 | RR 0.11 [0.08, 0.20] | | Moderate | |
| **CRT at 3 months**  1 RCT | | Not serious | Not serious | | Serious^&^ | | Serious | Undetected | | Yes | IVC | | Sham injection | MD -83.29, [-125.92, -40.67] | | Low | |
| **CRT at 12 months**  1 RCT | | Not serious | Not serious | | Serious^&^ | | Serious | Undetected | | Yes | IVC | | Sham injection | MD 4.08, [-19.75, 47.90] | | Low | |
| **CRT at 3 months**  2 RCTs | | Not serious | Not serious | | Serious^&^ | | Serious | Undetected | | Yes | IVC, n=97 | | Conservative treatment, n=97 | MD -18.90, [-37.08, -0.73] | | Low | |
| **CNV area at 3 months**  2 RCTs | | Serious^*^ | Not serious | | Serious^&^ | | Serious | Undetected | | Yes | IVC, n=64 | | IVR, n=64 | MD -1.26, [-1.59, -0.93] | | Very Low | |
| **CRT at 1 month**  2 RCTs | | Not serious | Not serious | | Serious^&^ | | Serious | Undetected | | Yes | IVC, n=75 | | IVT, n=75 | MD -89.35, [-102.71, -72.99] | | Low | |
| **CRT at 3 months**  2 RCTs | | Not serious | Not serious | | Serious^&^ | | Serious | Undetected | | Yes | IVC, n=72 | | IVT, n=68 | MD -102.99, [-131.60, -74.37] | | Low | |
| **Safety** | | | | | | | | | | | | | | | | | |
| **subconjunctival hemorrhage**  2 RCTs | | Serious^*^ | Not serious | | Not serious | | Serious | Undetected | | Yes | IVC, 5/78 | | IVR, 4/79 | RR 1.26 [0.36, 4.43] | | Low | |
| **ocular hypertension**  2 RCTs | | Serious^*^ | Not serious | | Not serious | | Serious | Undetected | | Yes | IVC, 9/78 | | IVR, 9/79 | RR 1.00 [0.44, 2.31] | | Low | |
| **subconjunctival hemorrhage**  3 RCTs | | Not serious | Not serious | | Not serious | | Serious | Undetected | | Yes | IVC, 2/125 | | IVT, 1/120 | RR 1.35 [0.27, 6.80] | | Moderate | |
| **ocular hypertension**  3 RCTs | | Not serious | Not serious | | Not serious | | Serious | Undetected | | Yes | IVC, 3/117 | | IVT, 8/113 | RR 0.37 [0.10, 1.35] | | Moderate | |
| **DME** | | | | | | | | | | | | | | | | | |
| **Efficacy** | | | | | | | | | | | | | | | | | |
| **BCVA improvement at 1 month**  3 RCTs | | Not serious | Not serious | | Not serious | | Serious | Undetected | | Yes | IVC with LP, n=120 | | LP, n=120 | MD 0.04, [0.03, 0.06] | | Moderate | |
| **BCVA improvement at 3 months**  3 RCTs | | Not serious | Not serious | | Not serious | | Serious | Undetected | | Yes | IVC with LP, n=109 | | LP, n=110 | MD 0.09, [0.07, 0.11] | | Moderate | |
| **CMT at 1 month**  3 RCTs | | Not serious | Not serious | | Serious^&^ | | Serious | Undetected | | Yes | IVC with LP, n=120 | | LP, n=120 | MD -15.16, -25.31, -5.02] | | Low | |
| **CMT at 3 months**  4 RCTs | | Not serious | Not serious | | Serious^&^ | | Serious | Undetected | | Yes | IVC with LP, n=141 | | LP, n=141 | MD -24.51, [-35.55, -13.46] | | Low | |
| * The result of synthesis may be influenced by Zhang YJ 2018, which was characterized to have reporting bias. & All of the fundus examination values were considered to be indirect evidence. # The whole trials included in this review didn’t meet the requirements of calculated sample size.   - **High quality:** further research is very unlikely to change our confidence in the estimate of effect. - **Moderate quality:** further research is likely to have an important impact on our confidence in the estimate of effect and may change the estimate. - **Low-quality:** further research is very likely to have an important impact on our confidence in the estimate of effect and is likely to change the estimate. - **Very low quality:** we are very uncertain about the estimate. | | | | | | | | | | | | | | | | | |
| **eTable 6 Summary of similar topic systematic reviews** | | | | | | | | | | | | | | | | |  |
| **Indications** | **Published reviews** | | | | | | | | | | | | | | | |  |
| **AMD** | **Cui 2018** | | | | | **Wang 2018** | | | | | | **Zhang 2018** | | | | |  |
|  | **Included studies** | | | **Note** | | **Included studies** | | | **Note** | | | **Included studies** | | | **Note** | |  |
|  | Han 2015 | | | Diabetic retinopathy | | Cai and Peng 2016 | | | Retrospective study | | | Li YY 2017 | | | Full-text unavailable | |  |
|  | Zhu and Liu 2015 | | | non-RCT | | Cui 2018 | | | Retrospective study | | | Mei HY 2017 | | | non-RCT | |  |
|  | Ren 2016 | | | non-RCT | | Huang 2018 | | | Retrospective study | | | Song W 2016 | | | Included in our review | |  |
|  | Dong 2016 | | | non-RCT | | Li 2018 | | | non-RCT | | | Liu ZN 2016 | | | Full-text unavailable | |  |
|  | He 2015 | | | Included in our review | | Lv 2016 | | | non-RCT | | | Zhang HX 2016 | | | Incomplete data | |  |
|  | Song 2015 | | | non-RCT | | Niu 2016 | | | non-RCT | | | Liu R 2015 | | | Included in our review | |  |
|  | Liu 2015 | | | Included in our review | | Yang 2018 | | | non-RCT | | | Wang NF 2017 | | | non-RCT | |  |
|  | Niu 2016 | | | non-RCT | | Zhang and Zhao 2016 | | | Incomplete data | | | Lv P 2016 | | | non-RCT | |  |
|  | Zhao and Bai 2015 | | | Retrospective study | | Zhang 2017 | | | non-RCT | | | Zheng MW 2017 | | | non-RCT | |  |
|  | Jin 2016 | | | non-RCT | | Zhang and Bai 2017 | | | non-RCT | | | Wang XX 2015 | | | Full-text unavailable | |  |
|  | Zhang 2015 | | | non-RCT | | Zhao and Bai 2015 | | | Retrospective study | | | Qin MM 2016 | | | Included in our review | |  |
|  | Wang 2015 | | | non-RCT | | Zheng 2017 | | | non-RCT | | | Li L 2017 | | | non-RCT | |  |
|  | Liu 2016 | | | non-RCT | |  | | |  | | | Zhang X 2015 | | | non-RCT | |  |
|  |  | | |  | |  | | |  | | | Zhu Y 2017 | | | non-RCT | |  |
|  |  | | |  | |  | | |  | | | He XT 2015 | | | Included in our review | |  |
|  |  | | |  | |  | | |  | | | Han X 2017 | | | Included in our review | |  |
|  |  | | |  | |  | | |  | | | Pan XL 2017 | | | non-RCT | |  |
|  |  | | |  | |  | | |  | | | Yue JL 2017 | | | non-RCT | |  |
| **DME** | **Liu 2019** | | | | | **Sun 2020** | | | | | | **Wang 2020** | | | | |  |
|  | **Included studies** | | | **Note** | | **Included studies** | | | **Note** | | | **Included studies** | | | **Note** | |  |
|  | Zhang 2018 | | | non-RCT | | Chen 2016 | | | non-RCT | | | Liu 2019 | | | Retrospective study | |  |
|  | Guo 2018 | | | Retrospective study | | Xu and Rong 2017 | | | Full-text unavailable | | | Li 2019 | | | Retrospective study | |  |
|  | Chen 2016 | | | non-RCT | | Guo 2018 | | | Retrospective study | | | Chang 2016 | | | Retrospective study | |  |
|  | Ji 2018 | | | Retrospective study | | Zhang 2018 | | | non-RCT | | | Jiang 2017 | | | non-RCT | |  |
|  | Hou and Hu 2018 | | | non-RCT | | Hou and Hu 2018 | | | non-RCT | | | Guo 2018 | | | Retrospective study | |  |
|  | Xiang 2018 | | | non-RCT | | Xiang 2018 | | | non-RCT | | | Zhang 2018 | | | non-RCT | |  |
|  | Lin 2016 | | | non-RCT | | Jiang 2018 | | | Retrospective study | | | Sun 2018 | | | Inappropriate comparison | |  |
|  | Yang and Chen 2017 | | | non-RCT | | Ji 2018 | | | Retrospective study | | | Zhang 2018 | | | non-RCT | |  |
|  | Dong and Hou 2015 | | | non-RCT | |  | | |  | | | Ren 2019 | | | non-RCT | |  |
|  |  | | |  | |  | | |  | | | Li 2019 | | | Retrospective study | |  |
|  |  | | |  | |  | | |  | | | Zhang 2019 | | | Inappropriate comparison | |  |
|  |  | | |  | |  | | |  | | | Xu 2016 | | | Retrospective study | |  |
|  |  | | |  | |  | | |  | | | Qian 2017 | | | Retrospective study | |  |
|  |  | | |  | |  | | |  | | | Xu 2017 | | | Retrospective study | |  |
|  |  | | |  | |  | | |  | | | Li 2018 | | | Retrospective study | |  |
|  |  | | |  | |  | | |  | | | Li 2017 | | | Retrospective study | |  |
|  |  | | |  | |  | | |  | | | Niu and Ji 2018 | | | non-RCT | |  |
|  |  | | |  | |  | | |  | | | Xu 2019 | | | Retrospective study | |  |
|  |  | | |  | |  | | |  | | | Zhou 2019 | | | Retrospective study | |  |
|  |  | | |  | |  | | |  | | | Li 2019 | | | Inappropriate comparison | |  |
| **pmCNV** | No data | | | | | | | | | | | | | | | |  |
| There is not published review focusing on intravitreal conbercept (IVC) for the treatment of visual impairment due to pmCNV.  “Inappropriate comparison” means that the patients in trial group was prescribed as IVC with Chinese medicine, while the patients in control group use IVC monotherapy. | | | | | | | | | | | | | | | | |  |
